# Aggressive Behaviour of Metastatic Melanoma in a Patient with Neurofibromatosis Type 1

**DOI:** 10.1155/2015/431943

**Published:** 2015-03-29

**Authors:** Robert W. Foley, Robert M. Maweni, Aurelie Fabre, David G. Healy

**Affiliations:** ^1^School of Medicine and Medical Science, University College Dublin, Belfield, Dublin 4, Ireland; ^2^Conway Institute of Biomedical and Biomolecular Research, University College Dublin, Belfield, Dublin 4, Ireland; ^3^Department of Pathology, St. Vincent's University Hospital, Elm Park, Dublin 4, Ireland; ^4^Department of Cardiothoracic Surgery, St. Vincent's University Hospital, Elm Park, Dublin 4, Ireland

## Abstract

Malignant melanoma is a common skin neoplasm bearing poor prognosis when presenting with metastases. Rarely melanoma metastases present without an identifiable primary cutaneous lesion despite exhaustive workup. We describe the case of a solitary lung metastasis in a patient with neurofibromatosis type 1 without an identifiable primary tumour. The rapid progression of this malignant neoplasm that led to the patient's death within 1 year is described.

## 1. Case Presentation

**Figure 1:**
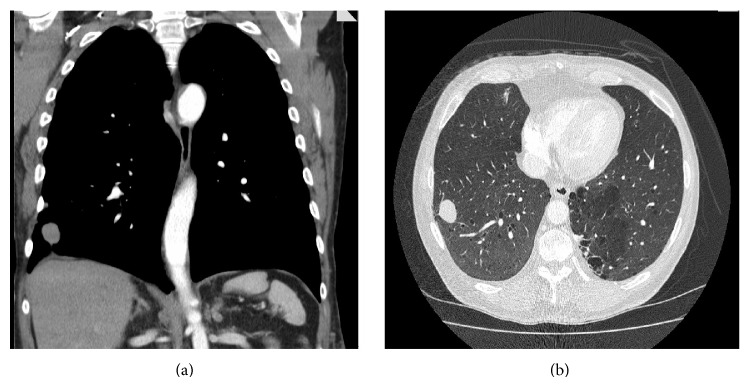
CT imaging of lung metastasis in coronal (a) and axial (b) planes.

**Figure 2:**
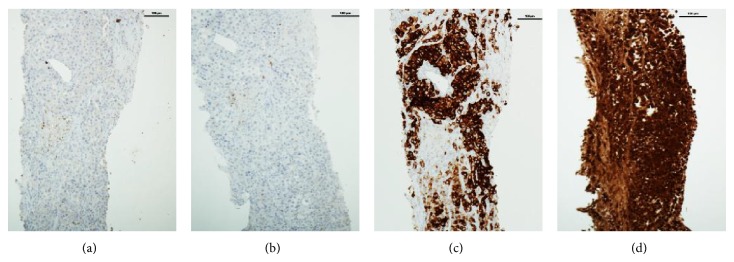
Histological analysis of lung biopsy. Tissue was negative for primary lung cancer ((a) CK stain and (b) TTF-1 stain) and was positive for melanoma ((c) Melan A stain and (d) S100 stain).

A 65-year old man with neurofibromatosis type 1 (NF1) underwent a chest X-ray following a three-week history of cough productive of white sputum. He was otherwise asymptomatic but had a 30-pack-year smoking history. The radiograph revealed a solitary coin lesion of the right lower lobe. The patient underwent a CT scan which demonstrated a 3.3 cm right lower lobe lesion, separate to the pleura and suspicious for primary lung carcinoma (Figure 1). A CT guided biopsy of the mass was obtained and histological examination demonstrated a deposit of large nucleolated epithelioid cells with pigment, positive for S100 and Melan A (Figure 2). The patient was discussed at a multidisciplinary meeting and a provisional diagnosis of metastatic melanoma was made.

Physical examination to establish the primary lesion was complicated by the presence of numerous neurofibromas and hyperpigmented café au lait spots, and the search ultimately proved unsuccessful. A PET/CT demonstrated a focal nodule in the right lung and several other masses with poor radiotracer uptake consistent with neurofibromas. Crucially the scan did not elicit a primary melanoma. The patient's ECOG performance status was 0 and therefore VATS metastasectomy with clear histological margins was performed. The tissue was stained for the BRAF V600 mutation but was negative and thus, the prescription of Vemurafenib was not deemed beneficial. Because the risk of systemic relapse was extremely high, the patient was followed with active surveillance.

**Figure 3:**
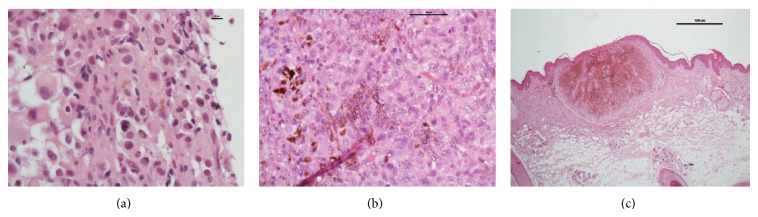
H&E stains on initial biopsy (a), metastasectomy (b), and primary melanoma excised from the neck (c).

The patient had undergone two surgical resections of large superficial plexiform neurofibromas 20 years ago. After the PET scan had proved negative, the possibility that these operations had removed the primary melanoma was considered. While attending the plastic surgery department 2 months after his VATS procedure for removal of an elbow neurofibroma, a pigmented lesion of the patient's skin, buried within his beard, was noted (Figure 3). Histologic analysis following excision demonstrated a superficial spreading malignant melanoma. It was Clark's level 4 tumour with a Breslow thickness of 1.6 cm. It was BRAF V600 mutation negative.

**Figure 4:**
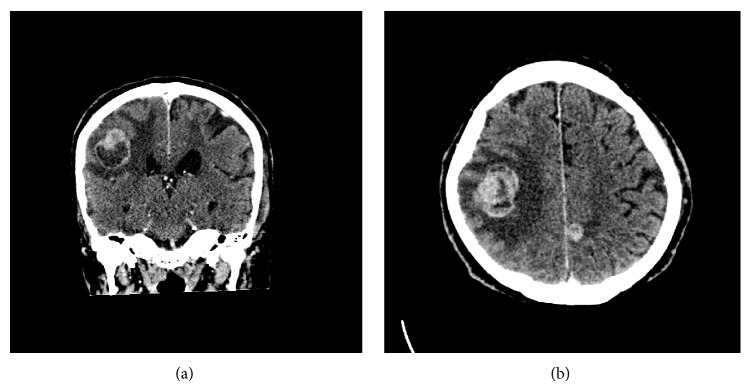
CT imaging of brain metastases in the coronal (a) and axial (b) planes.

One month after diagnosis, the patient suffered an acute stroke like episode, with subsequent CT brain indicating the presence of 6 intracranial lesions (Figure 4), compatible with metastases. The patient was treated with a course of whole brain radiotherapy and subsequently discharged to hospice care for palliative treatment. He succumbed to his illness 11 months after initial presentation, highlighting the rapid progression of this patient's malignant melanoma.

## 2. Discussion

NF1 patients are at increased risk of neural crest derived neoplasms [1]. Because melanocytes are derived from the embryologic neural crest, patients with NF1 have a propensity for developing this malignancy. The absence of an identifiable primary cutaneous, mucosal, or ocular melanoma in our patient raised the question of whether the tumour was a primary pulmonary melanoma or metastatic melanoma of unknown primary (MUP). Primary pulmonary melanoma is incredibly rare and is a controversial neoplasm due to unclear diagnostic criteria [2, 3]. Whereas MUP occurs in approximately 2.6% of patients presenting with metastasis, with lung second only to the lymphatic system as the most common site of occurrence [4, 5], MUP is associated with better outcomes and is generally less aggressive than non-MUP disease [6, 7].

Because melanoma is associated with late metastasis [8], it is a distinct possibility that this patient's metastasis originated from a primary cutaneous melanoma which had been removed incidentally. Conceivably during one of his previous surgeries. It is more likely, however, that the primary melanoma in this case was the lesion found in the patient's beard. Melanoma in NF1 is more commonly of orbital origin than the cutaneous lesion as described in this case [1]. Furthermore, cutaneous melanoma presenting with lung metastasis rarely proceeds to patient death within 1 year. Median survival in these patients is 17–50 months [9], emphasizing the rapidly progressive nature of the disease observed in our patient.

MUP should be investigated with PET/CT to locate the primary lesion and to stage the disease [10]. However the importance of a full physical examination, including oral cavity, cannot be overemphasized [11]. Similar to this case, however, it can take up to 8 months to locate the primary melanoma [12]. It is hypothesized that some lesions diagnosed as primary pulmonary melanomas are, in fact, metastases from occult primary lesions which have undergone spontaneous regression.

Regardless of the origin of the metastasis in this case, the benefit of performing metastasectomy was twofold. Firstly, a definitive diagnosis of the mass could be made as the oligometastatic lesion could have been manifesting as part of our patient's complex syndrome (NF1), while the possibility of a sarcoma still existed. And secondly a survival advantage would be conferred to the patient. The literature suggests aggressive surgical treatment is warranted in such cases. For all patients with unknown primary melanomas, the 5-year survival rate for those undergoing surgical resection (38.8%) was significantly better than that for patients who received no treatment (19.6%) [13]. This was also endorsed by de Wilt et al., who demonstrated an increase from 22% to 42% in 5-year survival in patients with primary pulmonary melanoma undergoing metastasectomy, with a median survival of 32 months [14].

This case illustrates a primary cutaneous melanoma presenting with a solitary pulmonary metastasis in a patient with NF1. This case is unusual due to the rapid progression of this patient's disease. Bearing in mind the close association and causative relationship of NF1 and malignant melanoma, a thorough physical examination of the patient who presents with a metastatic deposit of melanoma must be carried out in an effort to identify the primary lesion and to expedite the appropriate management strategy.
